# Supplementary material for: Complexome profiling of the Chlamydomonas psb28 mutant reveals TEF5 as an early PSII assembly factor
Source: Plant Cell. 2025 Mar 17;37(6):koaf055. doi: 10.1093/plcell/koaf055 (PMC12164590; doi:10.1093/plcell/koaf055)
Supplement: koaf055_Supplementary_Data [file koaf055_supplementary_data.zip › psb28_interactive.zip!/plots/intensity/qualityPlots/peptides/_Cre10.g439850.t1.2.html]

### Cre10.g439850.t1.2

Trivial name:   
SeqCoverage [median]: 19.90  
Mass: 73.12 kDa  
  
 

| Sequence | MissedCleavage | Mass [Da] | wtIntensity | muIntensity | Score |
| --- | --- | --- | --- | --- | --- |
| AKVEDDYRVNMILDNLPVAMVK | 2 | 2532.31 | 0.00 | 2701.39 | 53.55 |
| ASLEGQTEVK | 0 | 1060.54 | 4016.54 | 11755.34 | 119.68 |
| DAQTDLAR | 0 | 888.43 | 2353.92 | 6048.10 | 98.52 |
| DESTGSLVK | 0 | 934.46 | 1357.91 | 2342.18 | 67.03 |
| DVLFLK | 0 | 733.44 | 632.60 | 0.00 | 74.83 |
| FTILYHK | 0 | 920.51 | 0.00 | 1629.84 | 92.47 |
| FTILYHKDAQTDLAR | 1 | 1790.93 | 664.34 | 1110.97 | 50.16 |
| GDRIENSLYQIQMR | 1 | 1721.85 | 916.17 | 2698.74 | 73.39 |
| GFPVGFK | 0 | 750.41 | 553.52 | 0.00 | 74.60 |
| IENSLYQIQMR | 0 | 1393.70 | 705.28 | 1469.78 | 148.32 |
| IESLSSAQAK | 0 | 1032.55 | 10045.79 | 20931.70 | 140.14 |
| IVGFEVEPFSVK | 0 | 1349.72 | 787.57 | 4644.25 | 78.65 |
| IVQSAENLGEVLR | 0 | 1426.78 | 6138.04 | 20943.02 | 206.36 |
| IVQSAENLGEVLRGDR | 1 | 1754.93 | 4640.66 | 10829.13 | 57.16 |
| KDESTGSLVK | 1 | 1062.56 | 323.99 | 0.00 | 73.98 |
| MRKDESTGSLVK | 2 | 1349.70 | 3363.59 | 4912.14 | 68.03 |
| TNKIPR | 1 | 727.43 | 1113.97 | 0.00 | 137.44 |
| VNMILDNLPVAMVK | 0 | 1555.85 | 610.11 | 1020.87 | 118.48 |
|  |  |  | 38224.0 | 93037.4 |  |
